# A Case Report of Germline Compound Heterozygous Mutations in the *BRCA1* Gene of an Ovarian and Breast Cancer Patient

**DOI:** 10.3390/ijms22020889

**Published:** 2021-01-17

**Authors:** Ava Kwong, Cecilia Y. S. Ho, Vivian Y. Shin, Chun Hang Au, Tsun Leung Chan, Edmond S. K. Ma

**Affiliations:** 1 Department of Surgery, The University of Hong Kong and University of Hong Kong-Shenzhen Hospital, Hong Kong, China; vyshin@hku.hk; 2 Department of Surgery, Hong Kong Sanatorium & Hospital, Hong Kong, China; 3 Hong Kong Hereditary Breast Cancer Family Registry, Hong Kong, China; chris.tl.chan@hksh.com (T.L.C.); eskmca@hksh.com (E.S.K.M.); 4 Department of Molecular Pathology, Hong Kong Sanatorium & Hospital, Hong Kong, China; cecilia.ys.ho@hksh.com (C.Y.S.H.); tommy.ch.au@hksh.com (C.H.A.)

**Keywords:** hereditary breast cancer, compound heterozygous mutations, Chinese, Fanconi anemia

## Abstract

The germline carrier of the *BRCA1* pathogenic mutation has been well proven to confer an increased risk of breast and ovarian cancer. Despite *BRCA1* biallelic pathogenic mutations being extremely rare, they have been reported to be embryonically lethal or to cause Fanconi anemia (FA). Here we describe a patient who was a 48-year-old female identified with biallelic pathogenic mutations of the *BRCA1* gene, with no or very subtle FA-features. She was diagnosed with ovarian cancer and breast cancer at the ages of 43 and 44 and had a strong family history of breast and gynecological cancers.

## 1. Introduction

Hereditary breast and ovarian cancer syndrome is an inherited cancer-predisposition syndrome and is predominantly caused by mutations in the *BRCA1* or *BRCA2* genes. At least one functional allele of *BRCA1* is essential for human embryogenesis and development. Biallelic mutations in *BRCA1* were considered to be lethal during embryonic development [1], which was consistent with the observation in *BRCA1* homozygous knock-out mice models [2,3,4,5]. BRCA1 functions in the DNA double-strand break repair by interacting with other proteins such as RAD51 in the repair process. BRCA1 also interacts with proteins such as MSH2 in the DNA mismatch repair mechanism, and, possibly, poly (ADP-ribose) polymerase (PARP) in the single-strand repair [6]. Women carrying *BRCA1* pathogenic variant have a significant life time risk of breast and ovarian cancers as high as 84% [7]. Men with these mutations have an increased life time risk of breast cancer by 1% over male non-carriers [8], in addition to the risk of developing prostate carcinoma. Other studies reported that significant risk of melanoma and pancreatic cancer was observed in *BRCA* mutation carriers [9,10]. *BRCA1* mutations have also been linked to increase risk in cervical, esophagus, liver, stomach, and uterine cancers; however, the increased risks ranged from one to four fold and the exact risk was inconsistent [11,12,13,14].

*BRCA1* biallelic pathogenic mutations are extremely rare, which are regarded as embryonically lethal [1] or causing Fanconi anemia (FA) [15]. FA occurs in 1 in 160,000-360,000 individuals worldwide [16]. It has a high incidence in individuals of Ashkenazi Jewish descent, the Roma population of Spain and the Afrikaner population of South Africans [17]. FA is a rare genetic disease with multi-organ disorder and patients usually have variable clinical presentations. About 90% of the FA patients have an impaired bone marrow function that leads to aplastic anemia, frequent infections and clotting problems. Meanwhile, 60% of the FA patients have physical abnormalities, such as short stature, microcephaly and a triangular face. Other congenital abnormalities include malformed or absent kidneys, gastrointestinal abnormalities, heart defects, eye abnormalities and hearing loss [18]. The likelihood of developing cancers in individuals with Fanconi anemia is between 10% and 30%, and studies showed that these individuals are hypersensitive to DNA crosslinking agents, such as mitomycin C and cisplatin [19]. Mutations in at least 22 genes resulted in FA or FA-like phenotype, since proteins that produced from these genes are involved in DNA replication and repair. Among the 22 FA or FA-like genes, *BRCA1* is considered as *FANCS* (Fanconi anemia gene), homozygous mutations of the *BRCA1* gene contributed to FA group S [20]. Hence, it is crucial to identify individuals for whom both parents had *BRCA1* mutations.

Clinically, we would expect that surviving patients who are compound heterozygous for deleterious mutations in *BRCA1* would have showed an early manifestation of prominent FA features, such as anemia, infection tendency and cancer development [21,22,23]. Here, we described a family with a patient harboring two deleterious *BRCA1* mutations with no or very subtle FA-features.

## 2. Case Presentation

At the age of 43, the patient was first diagnosed with poorly differentiated high-grade serous adenocarcinoma of the ovary (stage IIIA), presented with abdominal distension for 1 month and was managed by optimal debulking surgery followed by post-operation paclitaxel and carboplatin (TC) regimen. She had right breast cancer with surgery 7 months later, showing pT1cN3M0 triple-negative invasive ductal carcinoma. She underwent lumpectomy which was followed by simple mastectomy with axillary lymph node dissection. She received adjuvant chemotherapy with 4 cycles of doxorubicin and cyclophosphamide, followed by local-regional radiotherapy up to 66 Gy and docetaxel. The patient developed extensive right chest wall recurrence one year after the mastectomy. She had mixed, but generally poor responses to chemotherapy regimes that escalated from the first line capecitabine. Her condition deteriorated in six months from the time of first recurrence, and progressed to extensive lymphadenopathy, pleural effusion, lung metastasis, liver and urinary bladder metastasis. She was also accidentally found to have an atrophic kidney and renal stone. Second line chemotherapy with 6 cycles of carboplatin (AUC4) and gemcitabine was given, restaging PET with extensive soft tissue metastasis and local recurrence was suggested. Third line chemotherapy with eribulin showed a partial response and eventually changed to fourth line chemotherapy, consisting of weekly doses of vinorelbine and carboplatin (AUC2). She subsequently had a trial of pembrolizumab and paclitaxel, though unfortunately she developed obstructive jaundice and clinically sepsis succumbed at four years after the initial ovarian surgery. No definitive diagnostic test for FA was performed on her due to a lack of clinical indication of FA-like features being observed. Her height was within the average Asian female range (165 cm; mean 157.1 cm [24]) with a normal complete blood picture. No hypersensitive response to carboplatin was noted during her treatment.

**Figure 1:**
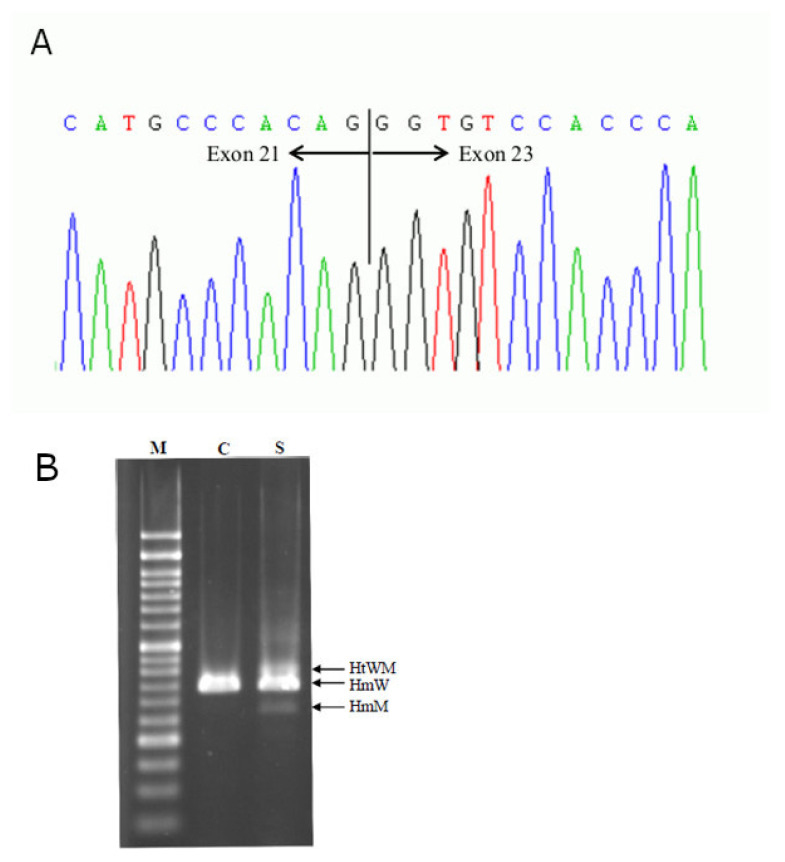
(**A**) Electropherogram showing that deletion of exon 22 on *BRCA1* c.5406+7A>G splicing. Deletion was detected by cDNA sequencing, (**B**) Gel electropherogram showing that deletion of 74bp by RT-PCR on *BRCA1* c.5406+7A>G. Lane M: 50-bp DNA ladder marker; lane C: Normal human cDNA control; lane S: Proband’s cDNA. HtNM: Heteroduplexes of wild-type and mutant; HmW: Homoduplexes of wild-type; HmM: Homoduplexes of mutant.

This patient was recruited by the Hong Kong Hereditary Breast Cancer Family Registry due to her young onset of breast and ovarian cancer, who met the selection criteria for genetic testing for hereditary breast and ovarian cancer syndrome. Her blood was screened by a next generation sequencing (NGS) four-gene panel that covered *BRCA1*, *BRCA2*, *TP53* and *PTEN*. Splicing variant analysis at the transcript level was performed by classic Sanger sequencing on the cDNA. Pathogenic mutations were further reconfirmed by Sanger sequencing. Compound heterozygotes *BRCA1* mutations were identified in this proband. One of the mutations was a deleterious deletion in exon 11 (c.4065_4068delTCAA; p.Asn1355Lysfs*10), while the other was a splice site mutation in intron 22 (c.5406+7A>G; r.5333_5406del74; p.Asp1778Glyfs*27). To further confirm the pathogenicity of c.5406+7A>G, we tested the cDNA from the blood of this patient, and confirmed a deletion of 74 nucleotides in her RNA. This deletion resulted in a frameshift termination after an amino acid substitution D1778G (r.5333_5406del74; p.Asp1778Glyfs*27) (Figure 1A,B). These findings confirmed the presence of alternative splicing. The mutation falls in one of the C-terminal BRCT domains of BRCA1, which was predicted to encode a dysfunctional BRCA1 protein.

**Figure 2:**
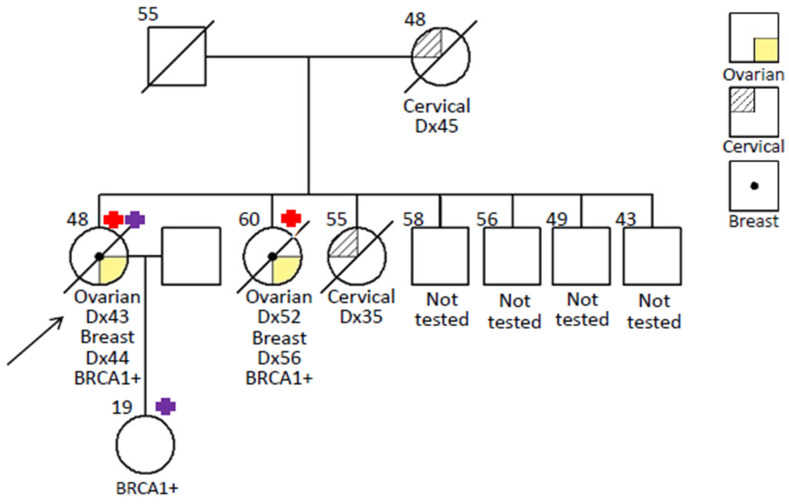
Pedigree of proband’s family. 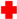: carrier of *BRCA1*: c.5406+7A>G; r.5333_5406del74; p.Asp1778Glyfs*27 mutations; 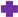: carrier of *BRCA1*: c.4065_4068delTCAA; p.Asn1355Lysfs*10.

In the family study, we were only able to obtain the genetic analyses on two of her family members. Her sister had ovarian cancer at the age of 52 and breast cancer at age 56. She carried only one of the two mutations of the proband, namely, *BRCA1*: c.5406+7A>G; r.5333_5406del74; p.Asp1778Glyfs*27. The unaffected cancer-free daughter aged 19 of the proband carried another mutation, *BRCA1*: c.4065_4068delTCAA; p.Asn1355Lysfs*10. These findings showed that the two mutations are in *trans* and the proband has biallelic mutations in *BRCA1* (Figure 2). This young girl has multiple benign breast lumps. Biopsies show benign breast tissue only. She is undergoing active clinical surveillance.

## 3. Discussions

Biallelic mutations in *BRCA1* are rarely reported. The occurrence of compound heterozygous mutations of *BRCA1* gene is very rare among breast cancer patients. Animal models predicted that such a genetic make-up would be a disadvantage to early embryonic development and survival [2,3,4,5]. In the literature, two adults and five consanguineous offspring at childhood had been reported (Table 1). These patients presented with cancers or congenital abnormalities. All these cases showed FA-like features in different degrees and penetrance. The short stature appeared to be a common presentation, and was clinically suspicious in some of these families. There were drastic differences in manifestations in all these patients, consistent with the classical description of the broad clinical spectrum of FA. This patient is the first Asian patient reported with a family study and with no significant FA features. This individual lack of physical stigmata with an average height and no dysmorphic features. A thorough examination was performed after confirmation of the biallelic *BRCA1* mutation. Subtle features, such as café-au-lait spots, were not seen. She was known to have an atrophic left kidney, pelvic stone and possibly renal vein thrombus, during clinical work-up for her first ovarian surgery. It was uncertain whether these abnormalities were acquired or congenital. Her clotting profile and complete blood picture before the first surgery and before all the subsequent chemo-radiotherapy were all unremarkable. She had no earlier imaging record to show any clinical significant urogenital problems. She had one daughter and did not indicate any fertility problem. Patients with FA are hypersensitive to DNA crosslinking agents, such as mitomycin C and cisplatin [9]. Such features were not obvious in this patient. There have been reports of individuals carrying biallelic *FANCM* mutations without developing FA; however, it has also been proved that *FANCM* is not a canonical FA gene [25,26].

**Figure 3:**
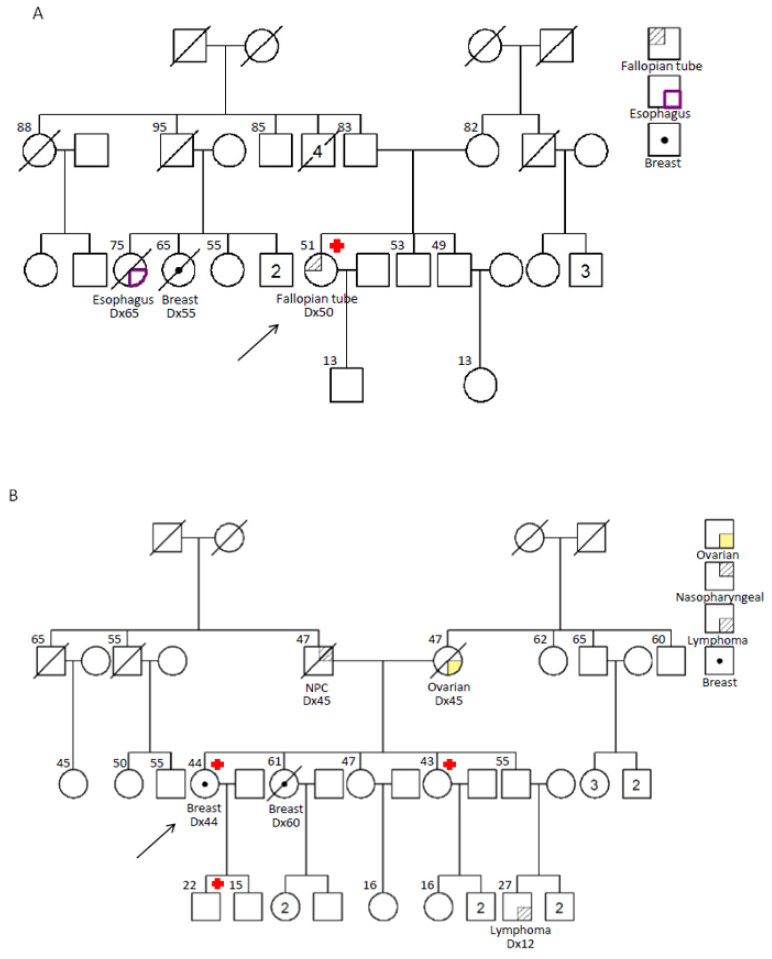
(**A**,**B**) Pedigree of the 2 families in our Hereditary Breast Cancer Family Registry with *BRCA1*: c.5406+7A>G; r.5333_5406del74; p.Asp1778Glyfs*27 mutations. 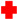: carrier of *BRCA1*: c.5406+7A>G; r.5333_5406del74; p.Asp1778Glyfs*27 mutations.

Among the two mutations identified in the proband, the deletion in exon 11 mutation, *BRCA1*: c.4065_4068delTCAA; p.Asn1355Lysfs*10, is well-documented. This variant is classified as a pathogenic variant in ClinVar (https://www.ncbi.nlm.nih.gov/clinvar/). The interpretation of the other mutation is more complex, it is a splice site variant in intron 22, c.5406+7A>G; r.5333_5406del74; p.Asp1778Glyfs*27. The ClinVar database of the National Library of Medicine, USA, shows that this variant as “likely benign” (referenced on 4^th^ Dec 2020), which is supported by four submissions based on germline studies. The associated comments suggest that this variant is a “conservative change”, as the variant occurs at a poorly conserved protein position. The variant has been predicted to be benign by multiple in silico algorithms (DANN: 0.5816; dbscSNV: 0.00002789 (ADA score)). The population frequency quoted in the ClinVar database was “not consistent with disease”. However, these submissions were from privately owned sequencing companies. Unfortunately, no specific details of the clinical evidencFe for these interpretations. In addition, this variant had not been reported in the Genome Aggregation Database (gnomAD; https://gnomad.broadinstitute.org/) in different populations. No splicing defect was observed in the functional studies [28]. This variant, in fact, cannot be considered as a common variant. We, therefore, proceeded to further characterize this variant. We confirmed that there was a deletion of 74 nucleotides in the cDNA of the patient’s blood and this deletion resulted in a frameshift termination in exon 22 (r.5333_5406del74; p.Asp1778Glyfs*27), as shown in Figure 1A,B. In addition to a previous study conducted by Khoo *et al*., this variant was also reported in Chinese breast and ovarian cancer patients and their protein truncation test has shown an early stop codon after splicing [29]. By integrating the genetic information and the family history, we could conclude that this mutation is pathogenic, which excluded the possibility of splice variants. Her sister with breast and ovarian cancers carried a c.5406+7A>G mutation, but did not carry the other, well-documented exon 11 deletion. The clinical features of her sister were compatible with predisposing effects of the deleterious *BRCA1* mutation. In our own Hereditary Breast Cancer Family Registry, we have additional evidence on the disease association of this intron 22 mutation. Two other unrelated families also carried this mutation (unpublished data). One of the families had a female proband with breast cancer diagnosed at the age of 44, suffering from pT1N0M0 ER/PR positive invasive ductal carcinoma, with a significant family history of breast cancers, ovarian cancers, nasopharyngeal cancers and lymphoma in her first and second-degree relatives (Figure 3A). Another family was a 51-year-old lady diagnosed with high-grade serous adenocarcinoma of the fallopian tube. There was also a strong family history of breast and esophagus cancers in her second-degree relatives (Figure 3B). Therefore, based on this clinical and laboratory evidence, we concluded that this intron 22 frame-shift mutation was pathogenic.

Usually patients carry biallelic deleterious mutations in cancer-causing genes are expected to exhibit prominent phenotypes and present with malignant diseases at a young age. The ages of onset of malignant diseases in this proband and her sister might have provided proof of this theory. Both proband and her sister had breast and ovarian cancers; however, the proband with two mutations had the conditions occur 10 years earlier than her sister, who carried only one mutation. Unfortunately, there was no genetic information about the four brothers of the proband. We therefore expected that they are likely to be carriers of *BRCA1* pathogenic variant. As there are definitive health risks for carriers and their family members, there is a need for better community education on the hereditary cancer risk of *BRCA1/2*, including for males. Studies showed that males had a low awareness of the *BRCA* mutation and were less willing to undergo genetic testing until their family members were identified to have a mutation [30,31]. Male relatives were less likely to be informed of test results and were more likely to forget about hearing them [27]. As exemplified by this family, male family members were reluctant to be subject to genetic testing. In our Registry’s data, 6.8% of all male family members compared with 10.4% of female family members came forward for genetic testing when a positive mutation was identified in their respective probands. In general, when both parents are carriers of pathogenic *BRCA1* mutations, their offspring should have a 75% of chance carrying at least one copy of the pathogenic mutation; hence, the clinical burden of developing cancer is predictable. It is still challenging to convey the concept of cancer risk and the prevention strategies of hereditary diseases to the general public. A better community program to educate the community for both males and females is necessary. 

## 4. Conclusions

Compound heterozygosity for deleterious *BRCA1* mutations exacerbated cancer disease phenotypes. If both parents are carriers of pathogenic *BRCA1* mutations, then their offspring should have a 75% chance of carrying at least one copy of the pathogenic mutation. Identification of families that show biallelic inheritance of *BRCA1* mutations is important for precise genetic counseling and implementation of disease surveillance or prevention strategies.

## Figures and Tables

**Table 1:** List of probands reported with biallelic *BRCA1* mutations.

References	*BRCA1* Mutations	Patient Background	Phenotype Suggesting Fanconi Anemia (FA)	Family History
[21]	heterozygous carrier of mutations p.Asp821Ilefs*25 and p.Val1736Ala in trans	28 year old woman with ovarian cancer at age 28	- short stature - microcephaly - developmental delay - significant toxicity from chemotherapy	- Breast, ovarian, intestinal cancer in 1st—4th degree relatives
[20]	heterozygous carrier of mutations p.Ser198Argfs*35 and p.Arg1699Trp	woman with breast cancer at age 23	- multiple congenital anomalies - limb defects and dysmorphic features - consistent with a FA-like disorder	- Lung, stomach, skin, endometrium and ovarian cancer in 1st—2nd degree relatives
[22]	homozygous carrier of a *BRCA1* nonsense mutation in exon 11 p.Cys903*	2.5 year old girl (consanguineous offspring)	- severe short stature - failure to thrive - neurodevelopmental delay - congenital heart disease - facial dysmorphic features - severe chromosomal fragility - consistent with a FA-like disorder	- Breast cancer in 1st—3rd degree relatives
[23]	homozygously carrier of *BRCA1* nonsense mutations in exon 11 p.Trp372*	5 year old girl with T cell ALL at age 5 (consanguineous offspring)	- multiple congenital anomalies - limb defects and dysmorphic features - severe chromosomal fragility - consistent with a FA-like disorder	- Intestinal and urological cancer in 2nd and 3rd degree relatives
		6 year old girl (consanguineous offspring)	- failure to thrive - multiple congenital anomalies - limb defects and dysmorphic features - severe chromosomal fragility - consistent with a FA-like disorder	- Intestinal and urological cancer, in 2nd and 3rd degree relatives
	homozygously carrier of *BRCA1* nonsense mutations in exon 11 p.Leu431*	15.5 year old boy (consanguineous offspring)	- Height < 3% percentile - multiple congenital anomalies - Right undescended testis, adrenal insufficiency - severe chromosomal fragility - consistent with a FA-like disorder	- Uterine, esophageal, and lung cancer in 2nd and 3rd degree relatives
		7 year old girl with Neuroblastoma at age 2 (consanguineous offspring)	- Height < 3% percentile - multiple congenital anomalies - growth hormone deficiency - Heart defect - severe chromosomal fragility - consistent with a FA-like disorder	- Uterine, esophageal, and lung cancer in 2nd and 3rd degree relatives
[27]	heterozygous carrier of missense mutations p.Arg1699Gln and p.Cys61Gly	30 year old woman with invasive-ductal breast cancer at age 30	- Height < 5% percentile - hearing loss (right side) - celiac disease - dysmorphic features - consistent with a FA-like disorder	- Breast and prostate cancer in 2nd degree relatives
This study	heterozygous carrier of nonsense mutations p.Asn1355Lysfs*10 and p.Asp1778Glyfs*27	48 year old woman with ovarian cancer at age 43 and breast cancer at age 44	- No FA-like disorder	- Breast, ovarian and cervix cancer in 1st degree relatives

## Data Availability

The data presented in this study are available on request from the corresponding author. The data are not publicly available due to restrictions of patient privacy.

## References

[1] Goldgar D., Easton D.F., Deffenbaugh A.M., Monteiro A.N.A., Tavtigian S.V., Couch F.J. (2004). Integrated Evaluation of DNA Sequence Variants of Unknown Clinical Significance: Application to BRCA1 and BRCA2. Am. J. Hum. Genet..

[2] Gowen L.C., Johnson B.L., Latour A.M., Sulik K.K., Koller B.H. (1996). Brca1 deficiency results in early embryonic lethality characterized by neuroepithelial abnormalities. Nat. Genet..

[3] Liu C.-Y., Flesken-Nikitin A., Li S., Zeng Y., Lee W.H. (1996). Inactivation of the mouse Brca1 gene leads to failure in the morphogenesis of the egg cylinder in early postimplantation development. Genes Dev..

[4] Hohenstein P., Kielman M.F., Breukel C., Bennett L.M., Wiseman R., Krimpenfort P., Cornelisse C., Van Ommen G.-J., Devilee P., Fodde R. (2001). A targeted mouse Brca1 mutation removing the last BRCT repeat results in apoptosis and embryonic lethality at the headfold stage. Oncogene.

[5] Evers B., Jonkers J. (2006). Mouse models of BRCA1 and BRCA2 deficiency: Past lessons, current understanding and future prospects. Oncogene.

[6] Maresca L., Lodovichi S., Lorenzoni A., Cervelli T., Monaco R., Spugnesi L., Tancredi M., Falaschi E., Zavaglia K., Landucci E. (2018). Functional Interaction Between BRCA1 and DNA Repair in Yeast May Uncover a Role of RAD50, RAD51, MRE11A, and MSH6 Somatic Variants in Cancer Development. Front. Genet..

[7] Chen S., Iversen E.S., Friebel T., Finkelstein D., Weber B.L., Eisen A., Peterson L.E., Schildkraut J.M., Isaacs C., Peshkin B.N. (2006). Characterization of BRCA1 and BRCA2 Mutations in a Large United States Sample. J. Clin. Oncol..

[8] Tai Y.C., Domchek S., Parmigiani G., Chen S. (2007). Breast Cancer Risk among Male BRCA1 and BRCA2 Mutation Carriers. J. Natl. Cancer Inst..

[9] Mersch J., Jackson M.A., Park M., Nebgen D., Peterson S.K., Singletary C., Arun B.K., Litton J.K. (2015). Cancers associated with BRCA1 and BRCA2 mutations other than breast and ovarian. Cancer.

[10] Iqbal J., Ragone A.V., Lubinski J., Lynch H.T., Moller P., Ghadirian P., Foulkes W.D., Armel S., Eisen A.Z., Neuhausen S.L. (2012). The incidence of pancreatic cancer in BRCA1 and BRCA2 mutation carriers. Br. J. Cancer.

[11] Ford D., Easton D.F., Bishop D.T., Narod S.A., Goldgar D.E. (1994). Risks of cancer in BRCA1-mutation carriers. Breast Cancer Linkage Consortium. Lancet.

[12] Moran A., O’Hara C., Khan S., Shack L., Woodward E., Maher E.R., Lalloo F., Evans D.G.R. (2012). Risk of cancer other than breast or ovarian in individuals with BRCA1 and BRCA2 mutations. Fam. Cancer.

[13] Phelan C.M., Iqbal J., Lynch H.T., Lubinski J., Gronwald J., Moller P., Ghadirian P., Foulkes W.D., Armel S., Eisen A. (2014). Incidence of colorectal cancer in BRCA1 and BRCA2 mutation carriers: Results from a follow-up study. Br. J. Cancer.

[14] Thompson D., Easton D.F., Breast Cancer Linkage Consortium (2002). Cancer Incidence in BRCA1 Mutation Carriers. J. Natl. Cancer Inst..

[15] D’Andrea A.D. (2013). BRCA1: A missing link in the Fanconi anemia/BRCA pathway. Cancer Discov..

[16] Wu Z.H. (2013). The concept and practice of Fanconi Anemia: From the clinical bedside to the laboratory bench. Transl. Pediatr..

[17] Tipping A.J., Pearson T., Morgan N.V., Gibson R.A., Kuyt L.P., Havenga C., Gluckman E., Joenje H., De Ravel T., Jansen S. (2001). Molecular and genealogical evidence for a founder effect in Fanconi anemia families of the Afrikaner population of South Africa. Proc. Natl. Acad. Sci. USA.

[18] Silver H.K., Blair W.C., Kempe C.H. (1952). Fanconi syndrome; multiple congenital anomalies with hypoplastic anemia. AMA Am. J. Dis. Child..

[19] Kennedy R.D., D’Andrea A.D. (2005). The Fanconi Anemia/BRCA pathway: New faces in the crowd. Genes Dev..

[20] Sawyer S.L., Tian L., Kähkönen M., Schwartzentruber J., Kircher M., Majewski J., Dyment D.A., Innes A.M., Boycott K.M., Moreau L.A. (2015). Biallelic Mutations in BRCA1 Cause a New Fanconi Anemia Subtype. Cancer Discov..

[21] Domchek S.M., Tang J., Stopfer J., Lilli D.R., Hamel N., Tischkowitz M., Monteiro A.N., Messick T.E., Powers J., Yonker A. (2013). Biallelic Deleterious BRCA1 Mutations in a Woman with Early-Onset Ovarian Cancer. Cancer Discov..

[22] Freire B.L., Homma T.K., Funari M.F., Lerario A.M., Leal A.M., Velloso E.D., Malaquias A.C., Jorge A.A.L. (2018). Homozygous loss of function BRCA1 variant causing a Fanconi-anemia-like phenotype, a clinical report and review of previous patients. Eur. J. Med Genet..

[23] Seo A., Steinberg-Shemer O., Unal S., Casadei S., Walsh T., Gumruk F., Shalev S., Shimamura A., Akarsu N.A., Tamary H. (2018). Mechanism for survival of homozygous nonsense mutations in the tumor suppressor gene BRCA1. Proc. Natl. Acad. Sci. USA.

[24] (2017). Report of Population Health Survey 2014/15.

[25] Catucci I., Osorio A., Arver B., Neidhardt G., Bogliolo M., Zanardi F., Riboni M., Minardi S., Pujol R., Azzollini J. (2018). Individuals with FANCM biallelic mutations do not develop Fanconi anemia, but show risk for breast cancer, chemotherapy toxicity and may display chromosome fragility. Genet. Med..

[26] Findlay G.M., Daza R.M., Martin B., Zhang M.D., Leith A.P., Gasperini M., Janizek J.D., Huang X., Starita L.M., Shendure J. (2018). Accurate classification of BRCA1 variants with saturation genome editing. Nat. Cell Biol..

[27] Keupp K., Hampp S., Hübbel A., Maringa M., Kostezka S., Rhiem K., Waha A., Wappenschmidt B., Pujol R., Surrallés J. (2019). Biallelic germline BRCA1 mutations in a patient with early onset breast cancer, mild Fanconi anemia-like phenotype, and no chromosome fragility. Mol. Genet. Genom. Med..

[28] Khoo U., Ngan H.Y., Cheung A.N., Chan K.Y., Lu J., Chan V.W., Lau S., Andrulis I.L., Ozcelik H. (2000). Mutational analysis of BRCA1 and BRCA2 genes in Chinese ovarian cancer identifies 6 novel germline mutations. Hum. Mutat..

[29] McAllister M.F., Evans D.G., Ormiston W., Daly P. (1998). Men in breast cancer families: A preliminary qualitative study of awareness and experience. J. Med Genet..

[30] Ibrahim M., Yadav S., Ogunleye F., Zakalik D. (2018). Male BRCA mutation carriers: Clinical characteristics and cancer spectrum. BMC Cancer.

[31] Daly M., Montgomery S., Bingler R., Ruth K. (2016). Communicating genetic test results within the family: Is it lost in translation? A survey of relatives in the randomized six-step study. Fam. Cancer.

